# Tuning of Morphological and Antibacterial Properties of Poly(3,4-ethylenedioxythiophene):Peroxodisulfate by Methyl Violet

**DOI:** 10.3390/polym15143026

**Published:** 2023-07-12

**Authors:** Sonal Gupta, Udit Acharya, Muhammed Arshad Thottappali, Hana Pištěková, Zuzana Morávková, Jiřina Hromádková, Oumayma Taboubi, Jiří Pfleger, Petr Humpolíček, Patrycja Bober

**Affiliations:** 1 Institute of Macromolecular Chemistry, Czech Academy of Sciences, 162 06 Prague, Czech Republic; sonalgpt24@gmail.com (S.G.); acharya@imc.cas.cz (U.A.); thottappali@imc.cas.cz (M.A.T.); moravkova@imc.cas.cz (Z.M.); hromadkova@imc.cas.cz (J.H.); taboubi@imc.cas.cz (O.T.); pfleger@imc.cas.cz (J.P.); 2 Centre of Polymer Systems, Tomas Bata University in Zlin, 760 01 Zlin, Czech Republichumpolicek@utb.cz (P.H.); 3 Department of Lipids, Surfactants and Cosmetics Technology, Faculty of Technology, Tomas Bata University in Zlin, 760 01 Zlin, Czech Republic

**Keywords:** poly(3,4-ethylenedioxythiophene), methyl violet, antibacterial properties, conductivity, electrochemical activity

## Abstract

This study demonstrates a one-step synthesis of poly(3,4-ethylenedioxythiophene) (PEDOT) in the presence of the methyl violet (MV) dye. The structural properties of PEDOT:peroxodisulfate were studied using Raman and MALDI-TOF spectroscopies. The use of the MV dye in the polymerization process resulted in a change in the typical irregular morphology of PEDOT:peroxodisulfate, leading to the formation of spherical patterns. SEM and TEM analyses revealed that increasing the dye concentration can produce larger spherical aggregates probably due to the hydrophobic and *π*–*π* interactions. These larger aggregates hindered the charge transport and reduced the electrical conductivity. Interestingly, at higher dye concentrations (0.05 and 0.075 M), the PEDOT:peroxodisulfate/MV films exhibited significantly improved antibacterial activity against *Staphylococcus aureus* and *Escherichia coli*. Furthermore, the PEDOT:peroxodisulfate films with the incorporated MV dye exhibited a well-defined and repeatable redox behavior. The remarkable amalgamation of their optical, electrochemical and antibacterial properties provides the PEDOT:peroxodisulfate/MV materials with an immensely diverse spectrum of applications, including in optical sensors and medical devices.

## 1. Introduction

Conducting polymer-based materials are gaining enormous attention in various biomedical applications as agents in photothermal therapy [1], sensors [2], bio-actuators [3], contrast materials for magnetic resonance imaging [4], in drug delivery [5], tissue engineering [6], etc. However, such applications require materials with antimicrobial properties protecting them from any contamination due to the adhesion or colonization of surface by pathogenic microorganisms [7]. Such antibacterial properties can be achieved in hybrid composite materials, in which, for example, metal or metal oxides [8], biopolymers (cellulose, chitosan, etc.) [9,10] and carbon materials [11] are combined with conducting polymers. Conducting polymers have also attained considerable interest for their highly sensitive and selective fluorescence-sensing properties. Such properties were documented by Ramanavicius et al. for a polypyrrole-based immunosensor [12] and by Ayranci et al. for a polypyrrole/glucose-oxidase composite [13]. Polyaniline nanoparticles showed fluorescence sensing properties and antibacterial activities originated from a photothermal effect [14]. In a recent study, ferrite-incorporated polypyrrole microcapsules loaded with fluorescent Nile Red dye demonstrated their promising applications in magnetic resonance imaging and as contrast agents [15].

Poly(3,4-ethylenedioxythiophene) (PEDOT) and its composites are known for their several attractive properties, such as high electrical conductivity, easy synthesis and processability, electrochemical stability and biocompatibility, which make them suitable for biomedical applications [16,17]. Various studies have described the nontoxicity of PEDOT and its capability for cellular adhesion and cell proliferation facilitation, which are promising for potential medical applications. [18,19]. Electrochemically deposited PEDOT electrodes were widely applied in neural stimulation and recording [20]. Many attempts were made to enhance antibacterial properties of PEDOT. For example, the chitosan based PEDOT material provided antioxidant and antibacterial activity [9,21]. In another example, magnetite nanoparticles in the composition with PEDOT were deposited on a textile and imparted high conductivity, UV protection and antibacterial properties against *S. aureus*, showing thus potential application in smart textiles, sensors and other biomedical applications [8]. In a study reported by Triguero et al., combined effects of PEDOT and lysozyme were used to demonstrate the antimicrobial activity with possible application in bio-capacitors [22].

In combination with conducting polymers, organic dyes have been recognized as an important tool for tuning their electrical conductivity, morphology and electrochemical activity [23,24]. Conducting polymers prepared in the presence of various dyes has recently shown potential in medical applications. For example, polypyrrole prepared using Acid Blue or acriflavine hydrochloride dyes was tested for its cytotoxic activity and antibacterial properties [24,25].

**Figure 1:**
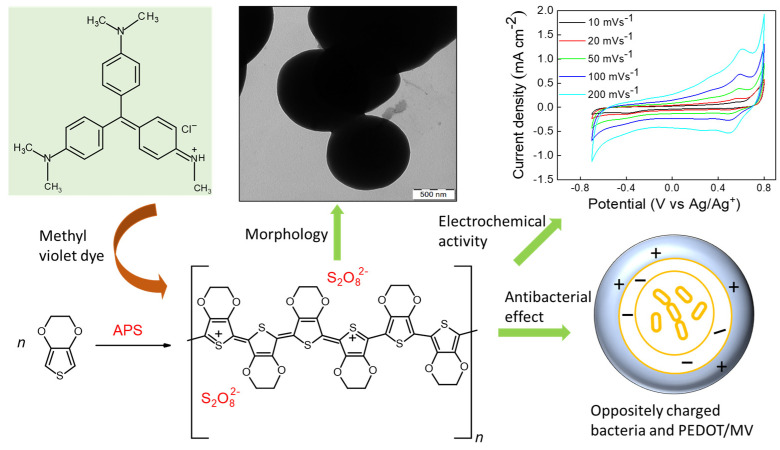
Schematic diagram of the synthesis of PEDOT using ammonium peroxodisulfate, structure of methyl violet and tunable properties of PEDOT.

In this article, we have extended our efforts to demonstrate the preparation of PEDOT material with the use of an inexpensive and antibacterial MV dye. This offers a cost-effective and environmentally friendly route to produce highly dispersible PEDOT:peroxodisulfate with spherical morphology, enhanced optical and electrochemical properties. However, the specific mechanism of interactions between PEDOT:peroxodisulfate and an MV dye is a complex research area that needs further research. The MV dye, also dubbed crystal violet, is well known for its medical uses [26], which has been a good motivation for testing its impact on possible antibacterial properties of PEDOT. The current work is focused on both the antibacterial activity and the optical properties of PEDOT tuned with the help of organic dye. Herein, we report the polymerization of EDOT using ammonium peroxodisulfate (APS) as an oxidant in the presence of the MV dye (Figure 1). The influence of dye concentration on the morphology of PEDOT:peroxodisulfate was studied using SEM/TEM. The optical properties of PEDOT:peroxodisulfate were analyzed using UV–vis absorption and PL spectroscopies. A MALDI TOF analysis was conducted to study the effect of the MV dye on the chain length of PEDOT:peroxodisulfate. Raman spectroscopy was employed to examine the presence of the MV dye and its effect on the chemical structure of PEDOT:peroxodisulfate. The thermal behavior of PEDOT:peroxodisulfate in the presence of the MV dye was evaluated using thermogravimetric analysis (TGA). Additionally, the role of the MV in the conductivity and electrochemical properties of PEDOT:peroxodisulfate was determined. Furthermore, the antibacterial activity of PEDOT with the incorporation of an inexpensive dye was accessed. The current work is focused on both the antibacterial activity and the optical properties of PEDOT:peroxodisulfate tuned with the help of organic dye which can enable new prospects for advanced material development for optical sensing and provide antibacterial properties.

## 2. Materials and Methods

### 2.1. Chemicals and Reagents

The 3,4-ethylenedioxythiophene (EDOT, 97%), ammonium peroxodisulfate (APS), methyl violet (MV, polymethylated pararosaniline hydrochlorides mixture) and Nafion 117 solution (lower aliphatic alcohols and water mixture) were purchased from Sigma Aldrich (St. Louis, MO, USA) and used without further purification. Carbon black (black pearls 2000) was supplied by Cabot (Clayton Australia).

### 2.2. Preparation of PEDOT with or without MV Dye

PEDOT was prepared by oxidation of 0.2 M EDOT using 0.25 M APS as an oxidant. EDOT was dissolved in 50 mL of methanol and APS was dissolved separately in 50 mL of distilled water. Then, APS solution was added to the EDOT solution keeping the fixed molar ratio of the APS to EDOT, [APS]/[EDOT] = 1.25. The mixture was shaken vigorously for 1–2 min, and then it was left undisturbed for 15 days at room temperature. The obtained product was filtered and washed with 0.2 M HCl and ethanol respectively. Finally, the solid product, PEDOT, was dried in a desiccator over silica gel to attain a constant weight.

For the synthesis of PEDOT in the presence of the MV, a series of MV concentrations from 0.005 to 0.075 M was used. The dye was solubilized in 50 mL of methanol together with EDOT and subjected to ultra-sonication for 15 min. The polymerization was carried out by adding an oxidant in a similar aforementioned procedure. It should be noted that some fraction of the dye was removed during washing with HCl and ethanol, as evidenced by the color of the supernatant and by the optical absorption spectra of the final product (see further in the text). The concentrations shown in the results refer, however, to the initial concentration of the dye in the reaction mixture. The yield (g g^−1^) of the PEDOT was calculated by taking the ratio between the weight of the obtained PEDOT and the amount of EDOT used.

### 2.3. Characterization

Morphological examination of powdered PEDOT was performed using a MAIA3 TESCAN scanning electron microscope (SEM; Oxford Instruments, Abingdon, UK) and a TEC-NAI G2 SPIRIT transmission electron microscope (TEM, FEI; Czech Republic).

The electrical conductivity was measured on compressed pellets (diameter 13 mm and thickness 1.0 ± 0.3 mm, prepared under pressure of 530 MPa with a hydraulic press Trystom H-62, Trystom, Czech Republic) using an Alpha-A Analyzer (Novocontrol Technologies, Germany) under applied AC voltage 1 V_rms_ in the frequency range 10^7^ to 10^−2^ Hz. The pellets were placed between the gold-plated brass disk electrodes in the ZGS active sample cell.

Thermal gravimetric analysis (TGA) was performed at a heating ramp rate of 10 °C min^−1^ at a fixed air flow rate of 25 mL min^−1^ from 25 to 800 °C using a Pyris 1 TGA thermogravimetric analyzer (Perkin Elmer, Shelton, CT, USA).

UV–vis absorption was acquired on a Lamda 950 UV–vis spectrometer (Perkin Elmer, Beaconsfield, UK). Photoluminescence (PL) emission spectra were obtained using FS5 (Edinburg Instruments, Edinburg, UK) spectrometer. For each absorption and emission measurement, the samples were dispersed in dimethylsulfoxide (DMSO, 10 mg mL^−1^) and diluted to ~400 times. The concentrations of the MV and PEDOT for the optical measurements were 0.06 mM and 0.10 mM, respectively.

Raman spectra were collected on a Renishaw inVia Reflex Raman microscope spectrometer (inVia Reflex, Renishaw, New Mills, UK) equipped with an objective with 50× magnification (Leica DM LM, Leica Microsystems, Wetzlar, Germany) and holographic grating 2400 lines mm^−1^ in combination with an Ar-ion 514 nm laser or holographic grating 1200 lines mm^−1^ in combination with a diode 785 nm laser excitation, respectively, at several different spots of each sample spread on a steel support.

Cyclic voltammetry was carried out using AUTOLAB (PGSTAT302N, Metrohm, Herisau, Switzerland) potentiostat. For each measurement, ~8 mg of finely crushed PEDOT with or without dye was mixed with 2 mg of carbon black and homogeneously dispersed in the mixture of 400 uL isopropanol, 590 uL milli Q water and 10 uL Nafion to make the total volume of 1 mL. Then, 1 uL of the prepared polymer dispersion was drop cast onto a glassy carbon (GC, diameter = 3 mm) under N_2_ atmosphere. GC, Ag/Ag^+^ and Pt were used as working, pseudo reference and counter electrodes, respectively, in a three-electrode electrochemical cell. As a supporting electrolyte, 0.2 M HCl was used. All the cyclic voltammetry studies were performed under N_2_ atmosphere.

For the antibacterial test, each sample was ground in a porcelain mortar to a fine powder, then weighed to prepare a stock concentrated suspension. From each sample, stock concentrated suspension was prepared in a sterile Mueller Hinton Broth (MHB) medium at a concentration of 64 mg mL^−1^ and the stock suspension was further diluted to concentrations of 32, 16, 8, 4, 2, 1, 0.5, 0.25, 0.125, 0.062 and 0.031 mg mL^−1^. An equal amount of bacterial suspension (inoculum) in MHB was added to each concentration and, after thorough homogenization, the tubes were incubated for 24 h at 35 °C. Then, 0.1 mL was taken from each tube and spread on the surface of the Trypton Soya Agar (in duplicate for each concentration). After incubation at 35 °C for 18 to 24 h, the growth of the test bacteria on the surface of the plates was evaluated and the minimum inhibitory concentration was determined accordingly. The tests were performed with *Staphylococcus aureus (S. aureus)* CCM 4516 (inoculum concentration 1.1×10^6^ CFU mL^−1^) and *Escherichia coli (E. coli)* CCM 4517 (inoculum concentration 1.1 × 10^6^ CFU mL^−1^).

## 3. Results and Discussion

### 3.1. Morphology

**Figure 2:**
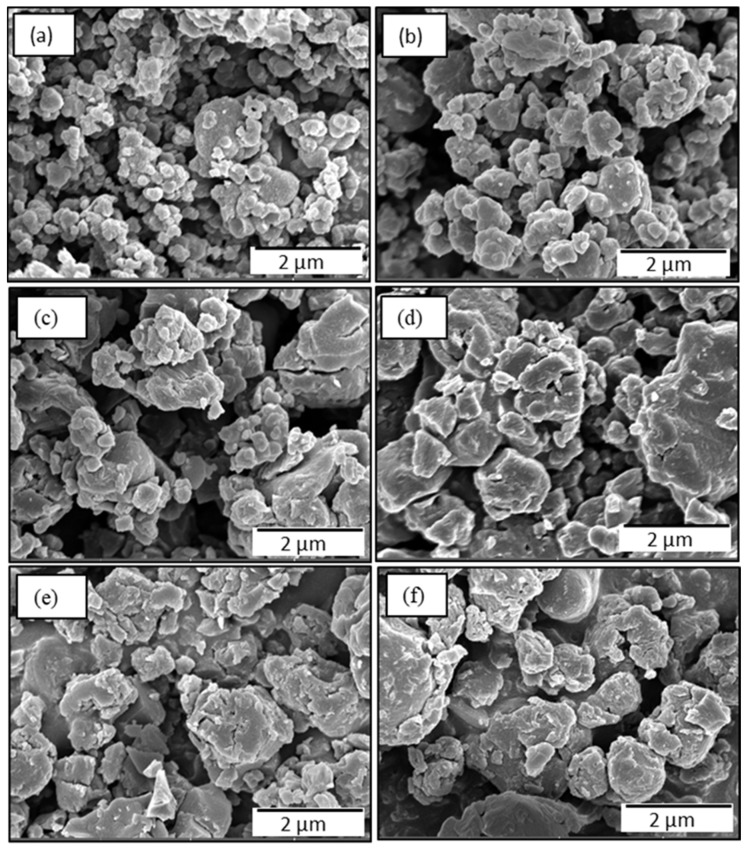
SEM images of PEDOT:peroxodisulfate prepared in the presence of (**a**) 0, (**b**) 0.005, (**c**) 0.01, (**d**) 0.02, (**e**) 0.05 and (**f**) 0.075 M of MV.

**Figure 3:**
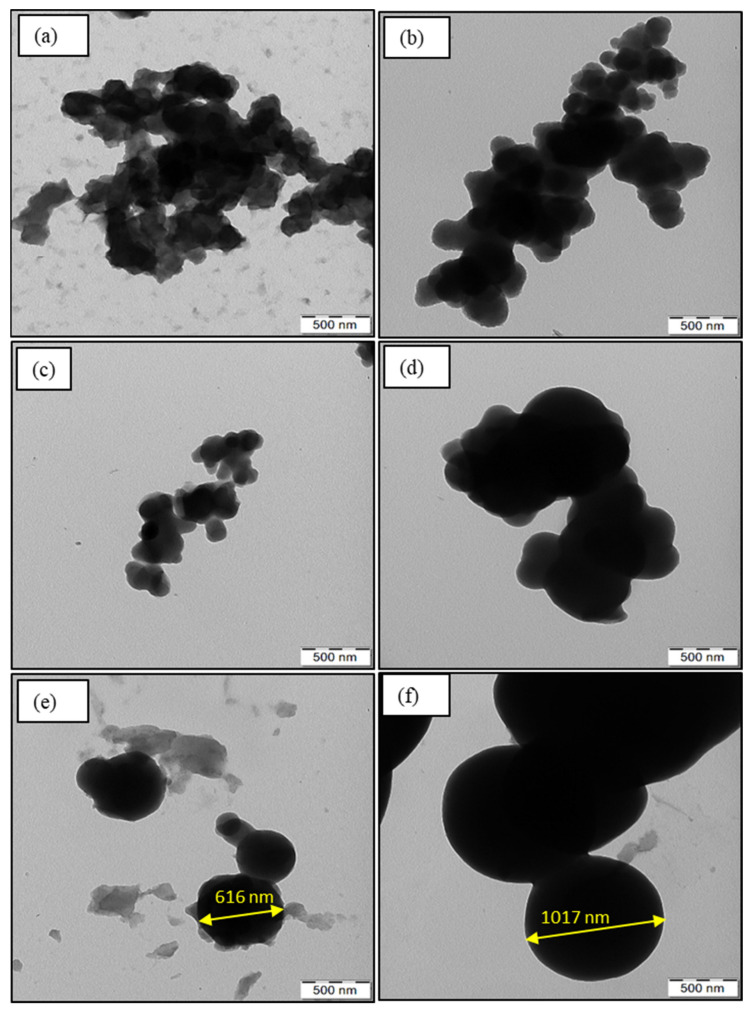
TEM images of PEDOT:peroxodisulfate prepared in the presence of (**a**) 0, (**b**) 0.005, (**c**) 0.01, (**d**) 0.02, (**e**) 0.05 and (**f**) 0.075 M of MV.

The oxidation of EDOT with APS produces PEDOT:peroxodisulfate in a form of a black powder containing particles of irregular shape (Figure 2a and Figure 3a), similar to the results reported earlier [27,28]. There have been already a few studies published that describe the influence of organic dyes on the morphological properties of PEDOT. Dai et al. demonstrated the synthesis of microtubular PEDOT using methyl orange as a template [29]. Bai et al. achieved the granular morphology of PEDOT along with the formation of channels by the help of Congo red [28]. In this report, we show that the addition of the MV to the polymerization mixture at increasing concentration, with fixed molar ratio of APS/EDOT, results in a gradual formation of spherical pattern (Figure 2b and Figure 3b). The particle size was observed to increase with the increasing dye content. The SEM/TEM micrographs (Figure 2c,d and Figure 3c,d) show the formation of spheres along with the preservation of some irregular pattern, which suggests the consumption of all the dye during polymerization. However, further increasing the dye concentration results in a higher number of spherical particles due to the adequate dye available for the polymerization and thus converting all irregular PEDOT:peroxodisulfate to dye-induced spheres (Figure 2e,f and Figure 3e,f). The TEM micrographs clearly show the diameter of a sphere in the range of 600 nm (Figure 3e), which can reach up to ~1100 nm with the further increase in the MV concentration (Figure 3f). The spherical morphology of PEDOT:peroxodisulfate/MV can be predicted by the interactions between the PEDOT:peroxodisulfate and MV, which in turn affects the optical properties of materials. The hydrophobic aromatic group of the MV tends to combine with EDOT molecules by π–π bonding and hydrophobic interactions [28].

### 3.2. Optical Properties

**Figure 4:**
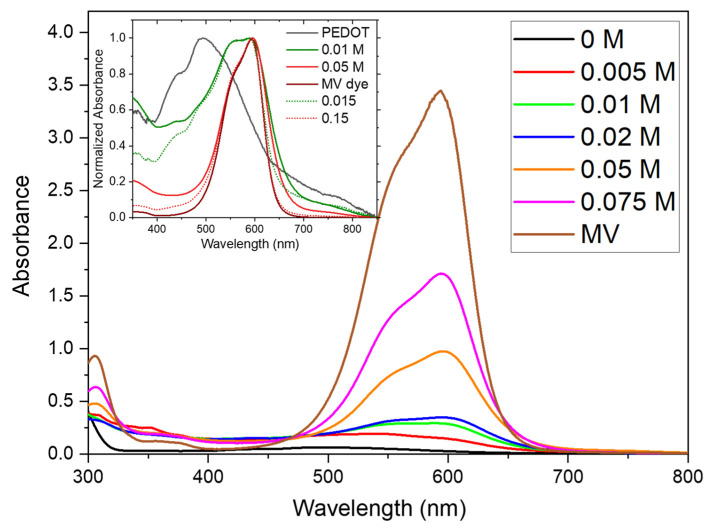
UV–vis absorption spectra of PEDOT:peroxodisulfate in the absence and in the presence of various concentrations of MV. Inset: Construction of the composite spectra (dotted lines) from the spectra of the pure components for 0.01 M and 0.05 M MV concentrations in the reaction mixture. Numbers in the legend show the real molar fraction of MV in the composites.

The investigation of optical properties, which were examined using UV–vis absorption and PL spectroscopies, play an important role in the fabrication of various optoelectronic devices. In order to examine the solution processability of PEDOT, solvents with different polarity were used. The polymers were found to be insoluble in hexane, chloroform, methanol and water. However, in high boiling solvents, such as the DMSO and *N*-methyl-2-pyrrolidone, PEDOT dispersions can be obtained. To understand the influence of the dye on the optical properties of PEDOT:peroxodisulfate, UV–vis absorption and PL spectroscopies were performed on samples dispersed in DMSO. The absorption spectrum of pristine PEDOT:peroxodisulfate presented in the inset of Figure 4 was found to be consistent with previously reported data [30]. Here, the broad absorption band ranging from 400 to 700 nm, with a maximum at 511 nm, corresponds to the π–π* transition [31], whereas the tail extending beyond 700 nm has been assigned to polaron and uncoupled bipolaron transitions [32]. The neat MV dye and the PEDOT:peroxodisulfate with higher MV concentration have the main peak located at 600 nm [33] with a shoulder around 560 nm originated in the optical transitions in the MV. The peak at 600 nm can be attributed to the molecularly dissolved dye, whereas the shoulder at 560 nm is related to the formation of H-aggregates of the dye [34]. With the dye concentration decreasing, the shoulder at 560 becomes suppressed and the PEDOT:peroxodisulfate band centered at 511 is increased. The H-aggregates refer to the ordered arrangements formed through the self-assembly of the MV molecules, where the dye molecules come together via intermolecular π–π stacking interactions. The presence of H-aggregates is evident from the sharp absorption band observed at 560 nm, and as the MV dye content increases in PEDOT:peroxodisulfate, there is a corresponding increase in the intensity of the absorption peaks at both 560 nm and 600 nm. The formation of H-aggregates implies a higher level of molecular organization and stacking within the PEDOT:peroxodisulfate, following the self-assembly and stacking of the MV dye molecules. There are unique properties possessed by H-aggregates such as shifted absorption peaks and enhanced light absorption in comparison to individual dye molecules. These characteristics are attributed to the unique organization and interactions of the aggregates. The presence of H-aggregates in the PEDOT:peroxodisulfate/MV reveals the presence of ordered assemblies of the MV dye molecules in the system, which in turn leads to tailored optical properties. [35,36]. As can be seen from the two examples in the inset of Figure 4, the composites spectra can be constructed from their two components. By fitting the composites spectra using their best ratio, the real fraction of the MV in the composites was determined and is shown in Table 1. This would not be otherwise possible due to the removal of the dye during product purification. Compared to simulated spectra, the real absorption bands are broader, and the absorbance rises towards the UV region, pointing to aggregate formation and corresponding to the increased light scattering. This is also in good agreement with the Raman results.

**Figure 5:**
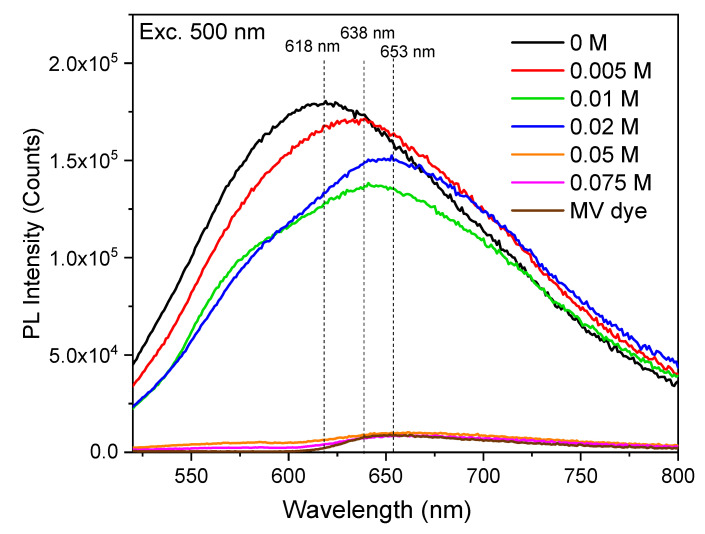
PL spectra of PEDOT:peroxodisulfate at excitation wavelengths of 500 nm in the absence and in the presence of various concentrations of MV.

The PL spectrum of PEDOT:peroxodisulfate shows a broad emission band in the visible region with the maxima at 618 nm when excited at 500 nm (Figure 5), which is in good agreement with a prior report [37]. The introduction of the MV results in an increase in the emission intensity for 0.005 M MV, which can be attributed to the easy charge recombination between the PEDOT:peroxodisulfate and the dye molecule. The MV at 0.01 and 0.02 M concentrations shows also the second peak at 638 nm, attributed to the MV. As suggested in the literature [38], the decrease in fluorescence intensity with the increase in dye concentration and the spectrum becoming similar to the dye itself at higher concentration suggests self-quenching as dye molecules pack closer or the formation of a non-fluorescent H-dimer at higher concentration. There is, however, a much more trivial explanation that the fluorescence quenching is caused by reabsorption of emitted light by the MV with much higher extinction coefficient and much lower fluorescence quantum yield.

### 3.3. MALDI TOF Spectra

**Figure 6:**
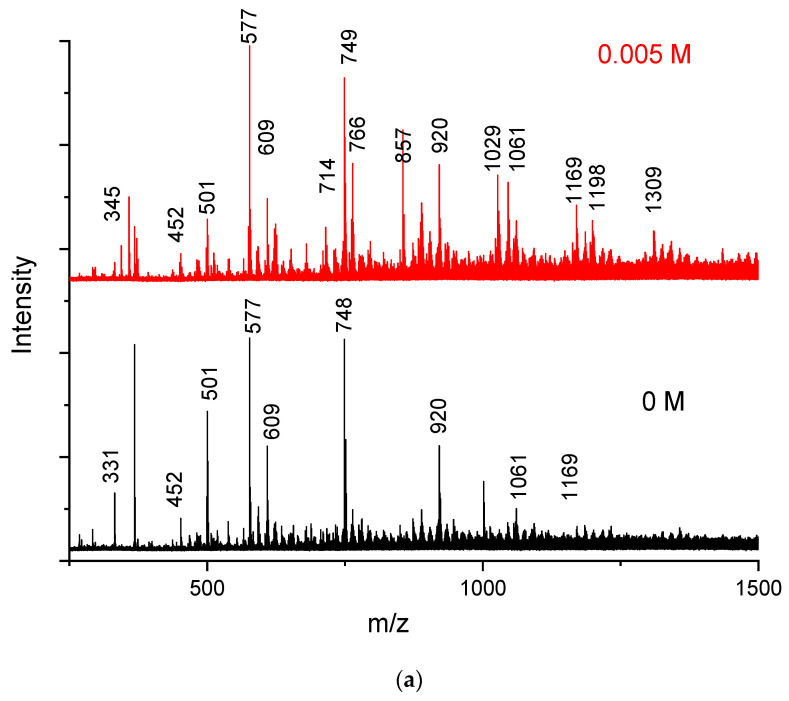

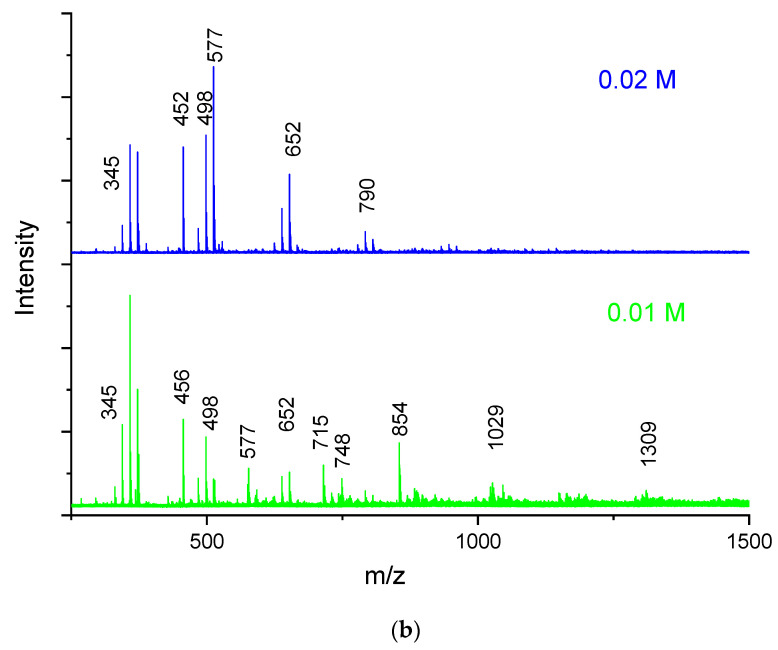
MALDI-TOF mass spectra of PEDOT:peroxodisulfate prepared in the presence of (**a**) 0 M and 0.005 M, (**b**) 0.01 M and 0.02 M MV dye.

To study the effect of the MV dye on the chain length of PEDOT:peroxodisulfate, the MALDI TOF analysis was performed. From the absorption or emission spectra, it was deduced that in a lower concentration of the MV, the properties of PEDOT:peroxodisulfate are comparable to or slightly better than those in pristine PEDOT:peroxodisulfate. The MALDI-TOF spectra of PEDOT:peroxodisulfate in the presence of 0.005, 0.01 and 0.02 M MV concentrations are shown in Figure 6. Pristine PEDOT:peroxodisulfate had the maximum molecular weight in the range of 1100–1200, indicating a polymer length of 7–8 repeating units. In the presence of 0.005 M and 0.01 M MV, PEDOT:peroxodisulfate had the highest molecular weight ~1300 (9–10 repeating units). However, a further increase in the dye concentration to 0.02 M did not increase the molecular weight of PEDOT:peroxodisulfate. When comparing all the MALDI-TOF spectra, PEDOT:peroxodisulfate prepared in the presence of the lowest dye concentration of 0.005 M shows the large intensity of peaks in the higher molecular weight region.

### 3.4. Yield and Conductivity

Previously, iron (III) chloride, cerium (IV) sulphate, iron (III) tosylate, etc., were used successfully as oxidants for EDOT polymerization. These oxidants act as counter ions to the positively charged PEDOT chain and also form a side product during the polymerization. As per the stoichiometry, six units of PEDOT are surrounded by two counter ions [39]. Following this, 1 g of EDOT yields 1.43 g of peroxodisulfate-doped PEDOT. The obtained yield for the PEDOT is 0.77 g g^−1^ (Table 1). Moreover, with the introduction of a small amount of dye, no significant change in the yield was observed. However, for the 0.005 and 0.075 M dye concentrations, a noticeable increase in the experimental yield was found.

**Figure 7:**
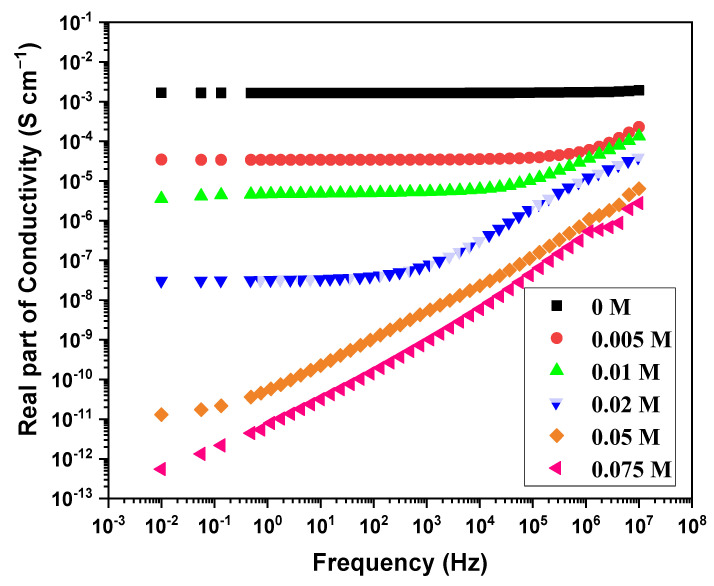
Frequency dependence of real part of conductivity.

The conductivity of PEDOT:peroxodisulfate decreases markedly with the increase in dye concentration (Table 1). The *π*–*π* interaction, ionic interaction, hydrogen bonding and hydrophobic interaction between the dye and the polymer chains are the most common interactions reported in the literature [40]. With the increase in dye concentration, the *π*–*π* interaction is dominated by the hydrophobic interaction which helps to form self-assembled H-aggregates, as evident in the TEM images. These large bulky aggregates with limited mobility show dispersive nature even at low frequencies (Figure 7). It is well known that dye resembles surfactants and aggregates to micelles, which are adsorbed on the polymer chains hindering the formation of the well-ordered arrangement of the conductive path [41].

### 3.5. Thermogravimetric Analysis

**Figure 8:**
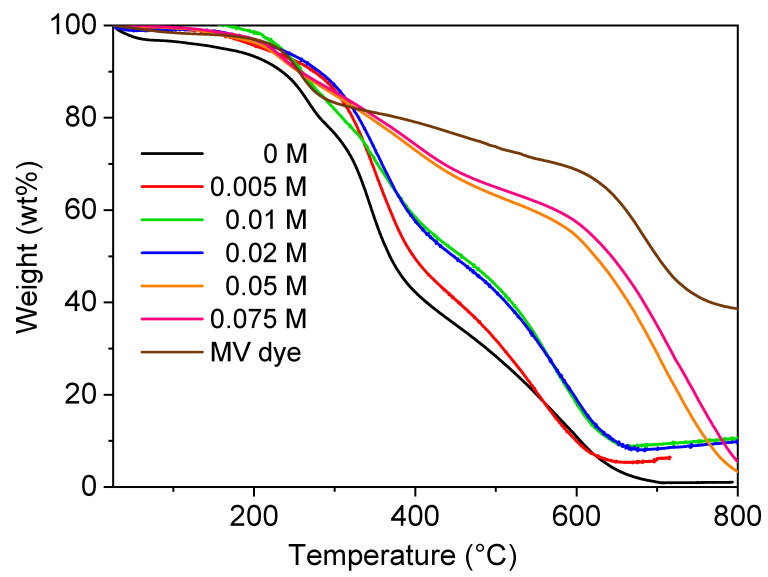
TGA in air of PEDOT:peroxodisulfate prepared with various MV concentrations.

The thermal behavior of PEDOT:peroxodisulfate in the presence of the MV dye was investigated (Figure 8). PEDOT:peroxodisulfate with or without dye shows stability up to 200 °C with 5–10 wt% loss corresponds to release of moisture content and protonating acid. The decomposition of PEDOT:peroxodisulfate is completed around 700 °C, whereas the PEDOT:peroxodisulfate in the presence of low concentrations of the MV, 0.005, 0.01 and 0.02 M, shows a residual weight of ~10 wt%. This can be associated with the neat MV, which itself displays the residue of ~37 wt%. Further, on increasing the concentration of MV to 0.05 and 0.075 M, PEDOT:peroxodisulfate exhibits complete decomposition. This variation in the thermal behavior with an increase in the amount of dye can be attributed to the different morphology of PEDOT:peroxodisulfate (Section 3.1), as the spherical morphology of the PEDOT:peroxodisulfate was obtained due to sufficient dye available in the polymerization mixture for high concentrations of the MV in comparison to the 0.05, 0.01 and 0.02 M concentrations where mixtures of irregular and spherical patterns were observed. The TGA analysis of the MV dye in the air atmosphere where the weight loss observed between 200 and 290 °C indicated the evolution of H_2_ and subsequent oxidation of hydrogen into H_2_O. Additionally, the weight loss observed between 300 and 600 °C was likely due to the oxidation of organic carbon and nitrogen, resulting in the production of CO_2_ and NO_2_ [42]. A previous study has reported residual weights of 30 wt% for methyl orange and 20 wt% for Acid Blue 25 in the TGA analyses of organic dyes. These residual weights likely consist of non-volatile components [43].

### 3.6. Raman Spectroscopy

**Figure 9:**
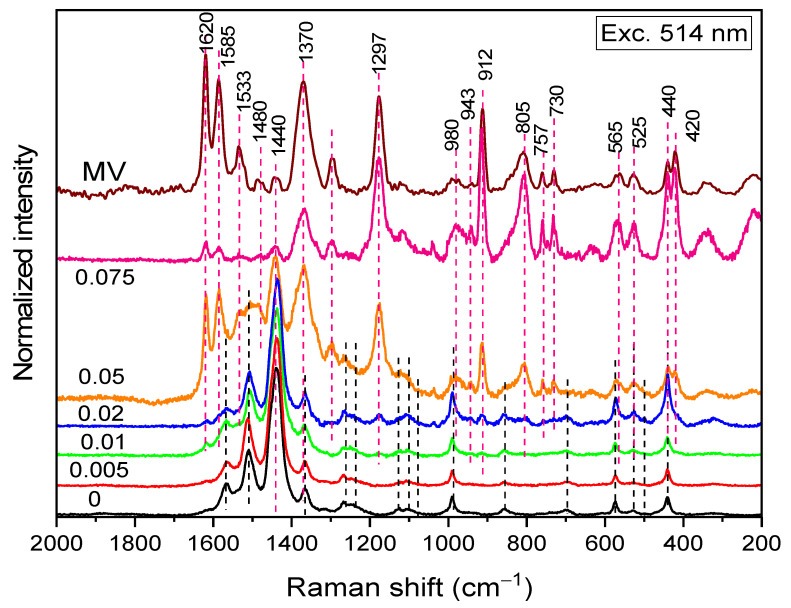
Raman spectra (excitation line 514 nm) of PEDOT:peroxodisulfate in the absence and in the presence of various concentrations of MV dye.

Raman spectra of PEDOT:peroxodisulfate prepared at various MV concentrations were measured with 514 nm excitation to detect the signal of the MV (Figure 9) (both MV and PEDOT Raman signal is (pre)resonantly enhanced, the enhancement being much stronger for MV). The dye has been detected at the lowest concentration of 0.01 M. The spectrum of the PEDOT:peroxodisulfate prepared at the highest dye concentration, 0.075 M, reflects mainly the MV with only minor peaks of PEDOT. No peak position shifts were observed; however, the C—C stretching band of the phenyl and quinonoid rings of MV at 1620 and 1585 cm^−1^, respectively, decreased in intensity dramatically. This is probably connected with the change in the resonance conditions in the composite compared to those in the neat dye (measured as a powder, i.e., with its molecules stacked).

**Figure 10:**
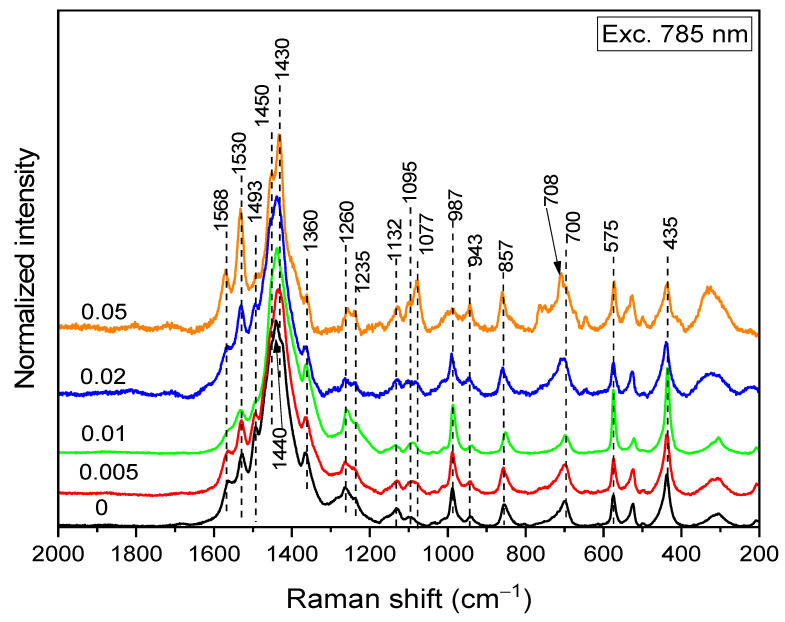
Raman spectra (excitation line 785 nm) of PEDOT:peroxodisulfate in the absence and in the presence of various concentrations of MV dye.

The Raman spectra excited with the 785 nm laser line reveal the state of the PEDOT:peroxodisulfate fraction in the composite (Table 2), as the MV dye is out of resonance with this line (Figure 10). The spectra of the samples with higher MV concentrations were, however, overlapped with MV fluorescence (and were thereby baseline corrected). The spectrum of PEDOT:peroxodisulfate prepared at the highest dye concentration, 0.075 M, was completely obscured by the fluorescence and is not presented. With increasing MV concentration, the intensity of the C=C stretching peak connected with the moderately-doped structures (polarons) at 1440 cm^−1^ decreases, while the intensities of the C=C stretching peak connected with both neutral (1530 and 1430 cm^−1^) and highly doped (bipolaron, 1450 cm^−1^) structures increase. Intensity changes were also observed for the bands connected with the oxyethylene ring, which also belongs to the charge-carrier delocalization system: the band at 1077 cm^−1^ increases, while the band at 987 cm^−1^ decreases. The maximum of the symmetrical C—S—C deformation band shifts from 700 to 708 cm^−1^. These changes indicate the coupling of the polarons into bipolarons induced by the elongation of the PEDOT:peroxodisulfate chains in the presence of MV.

### 3.7. Minimal Inhibitory Concentration

**Figure 11:**
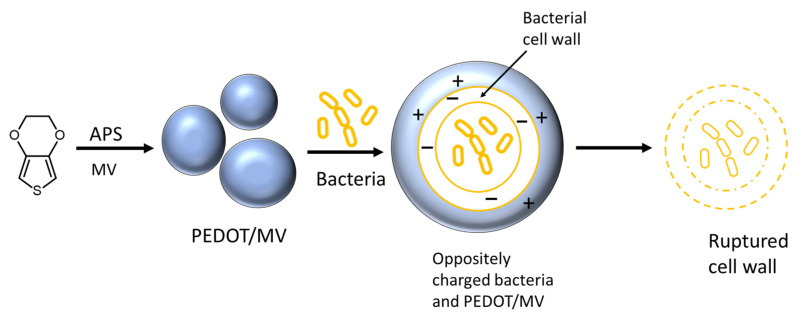
Antibacterial mechanism of PEDOT:peroxodisulfate prepared in the presence of MV.

The antibacterial activity was evaluated using the minimum inhibitory concentration (MIC) according to CLSI/EUCAST as shown in Table 3. The MIC is the lowest concentration in mg mL^−1^ of the antibacterial agent to prevent the bacterial growth under defined conditions. For our current study, we have presented the effect of the MV dye on antibacterial properties of PEDOT:peroxodisulfate against the two bacteria, i.e., *S. aureus* and *E. coli*. PEDOT:peroxodisulfate itself exhibits MIC of 16 mg mL^−1^ against the both bacteria. With the introduction of the MV dye, an increase in the MIC values was observed for lower dye concentration. However, when further increasing the dye concentration, i.e., at 0.05 and 0.075 M, an improvement in the antibacterial activity of the PEDOT:peroxodisulfate was found. The MIC values obtained showed that the PEDOT:peroxodisulfate in the presence of the dye is more effective against Gram-positive bacteria compared to Gram-negative. The PEDOT:peroxodisulfate with the largest amount of dye presented the best antibacterial effect against the two bacteria. The antibacterial mechanism is generally explained by the electrostatic forces acting between the conducting polymer and the bacterial cell wall. The positively charged antibacterial agents are responsible for the disruption of cell membranes, ultimately leading to the death of the bacteria (Figure 11) [52]. Previously published reports showed the enhanced antibacterial activity of PEDOT:peroxodisulfate by preparation of composites with inorganic or organic materials. For example, Sedighi et al. showed that a composite of PEDOT with Fe_2_O_3_ nanoparticles possessed antibacterial properties against the *S. aureus.* [8]. Kiristi et al. reported the effect of chitosan in improving the antibacterial properties in PEDOT [21]. In addition, Kumar and coworkers demonstrated its antibacterial activity using fluorohydroxyapatite nanoparticles [53]. Herein, we have assessed the antibacterial activity of PEDOT:peroxodisulfate with the incorporation of an inexpensive dye by a facile, one step procedure, which has not been reported so far.

### 3.8. Cyclic Voltammetry

**Figure 12:**
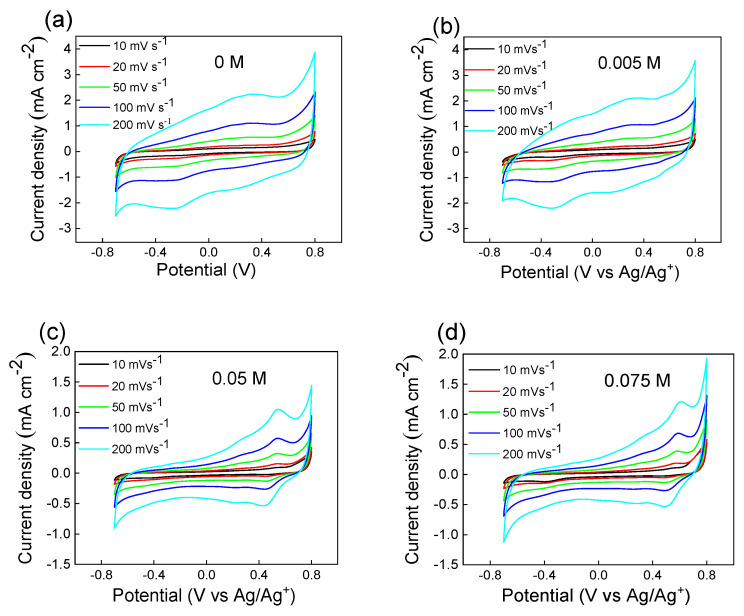
Cyclic voltammograms of PEDOT:peroxodisulfate in the presence of (**a**) 0, (**b**) 0.005, (**c**) 0.05 and (**d**) 0.075 M of MV.

Polymer films were prepared on a glassy electrode by a drop-casting method as explained in the experimental section. Figure 11 shows the cyclic voltammograms of PEDOT:peroxodisulfate in the absence and in the presence of 0.005, 0.05 and 0.075 M of MV. The various scan rates of 10, 20, 50, 100 and 200 mV s^−1^ were selected over the potential window of −0.7 to 0.8 V in 0.2 M HCl. When comparing the cyclic voltammograms, it was found that PEDOT:peroxodisulfate with the lowest MV dye concentration had fairly similar electroactivity to that of the pristine PEDOT (Figure 12a,b). When the other hand, in the presence of a higher amount of the MV, the influence of nonconducting dye on PEDOT:peroxodisulfate can be seen clearly (Figure 12c,d). The experiments were performed under various sweep rates to consider their electrochemical activity. It can be inferred that the films are quite stable and display well-defined redox behavior similar to previously reported [30]. The electrochemical activity of PEDOT:peroxodisulfate in the presence of various MV concentrations, with their promising fluorescence and antibacterial activity ensure their utility in optoelectronic and medical devices [54,55].

## 4. Conclusions

A one-step synthesis of PEDOT:peroxodisulfate in the presence of the MV dye, using APS, was demonstrated, resulting in spherical patterns with a size ranging from 600 to 1100 nm. A MALDI TOF analysis demonstrated that PEDOT:peroxodisulfate prepared at the lowest dye concentration of 0.005 M exhibits enhanced intensity in a high molecular weight region. The conductivity of PEDOT:peroxodisulfate decreases markedly from 10^–3^ to 10^–12^ S cm^−1^ with the increase in the MV concentration. The PEDOT:peroxodisulfate exhibited increased antibacterial effectiveness against the *S. aureus* bacteria, with MIC values of 0.125 mg mL^−1^ and 8 mg mL^−1^ at MV concentrations of 0.05 M and 0.075 M, respectively. The electrochemical activity of the PEDOT:peroxodisulfate with the incorporation of the MV dye was studied and attained well-defined redox behavior. The combination of PEDOT:peroxodisulfate with the organic dye not only provides tunable optical properties but also exhibits antibacterial activity, making it suitable for various applications in the medical field.

## Figures and Tables

**Table 1:** Yield, MV molar fraction and conductivity of PEDOT prepared in various molar concentrations of MV dye, [MV], in the reaction mixture with the fixed molar ratio of the APS to EDOT, i.e., [APS]/[EDOT] = 1.25. The molar fractions of MV in the polymer was obtained from modelling the optical absorption spectra.

[MV] (M)	Yield (g g^−1^)	Molar Fraction of MV in the Composite	Conductivity (S cm^−1^)
0	0.77	0	1.7 × 10^–3^
0.005	0.67	0.0048	3.4 × 10^–5^
0.010	0.74	0.015	4 × 10^–6^
0.020	0.77	0.024	3.1 × 10^–8^
0.050	0.95	0.15	1.5 × 10^–11^
0.075	1.4	0.30	9.5 × 10^–12^

**Table 2:** Raman frequencies assigned to PEDOT.

Position	Assignment	Reference
1568	asymmetrical C=C stretching in doped units	[44,45]
1530	asymmetrical C=C stretching in neutral units	[44,46,47,48]
1493	asymmetrical C=C stretching in doped units	[44,45,49]
1450	symmetrical C=C stretching in doped units	[44,45,49,50]
1440	symmetrical C=C stretching in doped units	[44,45,49]
1430	symmetrical C=C stretching in neutral units	[44,46,48,49,50]
1360	symmetrical C_β_—C_β_ stretching in neutral units	[44,45,46,47,48,49,50]
1260	symmetrical C_α_—C_α′_ stretching	[44,45,46,47,48,49]
1235	symmetrical C_α_—C_α′_ stretching in doped units and C—H deformation	[44,46,49]
1132	C—O stretching	[46,47,48,51]
1095	C—O—C deformation	[46,47,49,50]
1077	C—O—C deformation	[44,51]
987	oxyethylene ring	[45,46,47,49,50]
943	C—S deformation	[51]
857	oxyethylene ring, doped units	[44,47]
700/708	C—S—C symmetrical deformation	[45,46,47,48,49,50,51]
575	oxyethylene ring deformation	[45,46,47,49,50]
435	oxyethylene ring deformation	[46,47]

**Table 3:** Results of minimum inhibitory concentration of PEDOT:peroxodisulfate in the presence of various concentrations of MV dye.

Bacteria	Minimum Inhibitory Concentration (mg mL^−1^)					
	0 M	0.005 M	0.01 M	0.02 M	0.05 M	0.075 M
*S. aureus*	16	>32	>32	>32	0.5	0.125
*E. coli*	16	>32	>32	>32	16	8

## Data Availability

Data will be available upon request.
